# Urocortins in the mammalian endocrine system

**DOI:** 10.1186/s13028-019-0480-2

**Published:** 2019-10-04

**Authors:** Caterina Squillacioti, Alessandra Pelagalli, Giovanna Liguori, Nicola Mirabella

**Affiliations:** 1 Department of Veterinary Medicine and Animal Productions, University of Naples Federico II, Via Veterinaria 1, 80137 Naples, Italy; 2 Department of Advanced Biomedical Sciences, University of Naples Federico II, Via Pansini 5, 80131 Naples, Italy; 3 Institute of Biostructures and Bioimages, National Research Council, Via De Amicis 95, 80131 Naples, Italy

**Keywords:** Corticotropin-releasing hormone receptors, Endocrine system, Mammals, Urocortin

## Abstract

Urocortins (Ucns), peptides belonging to the corticotropin-releasing hormone (CRH) family, are classified into Ucn1, Ucn2, and Ucn3. They are involved in regulating several body functions by binding to two G protein-coupled receptors: receptor type 1 (CRHR1) and type 2 (CRHR2). In this review, we provide a historical overview of research on Ucns and their receptors in the mammalian endocrine system. Although the literature on the topic is limited, we focused our attention particularly on the main role of Ucns and their receptors in regulating the hypothalamic–pituitary–adrenal and thyroid axes, reproductive organs, pancreas, gastrointestinal tract, and other tissues characterized by “diffuse” endocrine cells in mammals. The prominent function of these peptides in health conditions led us to also hypothesize an action of Ucn agonists/antagonists in stress and in various diseases with its critical consequences on behavior and physiology. The potential role of the urocortinergic system is an intriguing topic that deserves further in-depth investigations to develop novel strategies for preventing stress-related conditions and treating endocrine diseases.

## Background

**Fig. 1:**
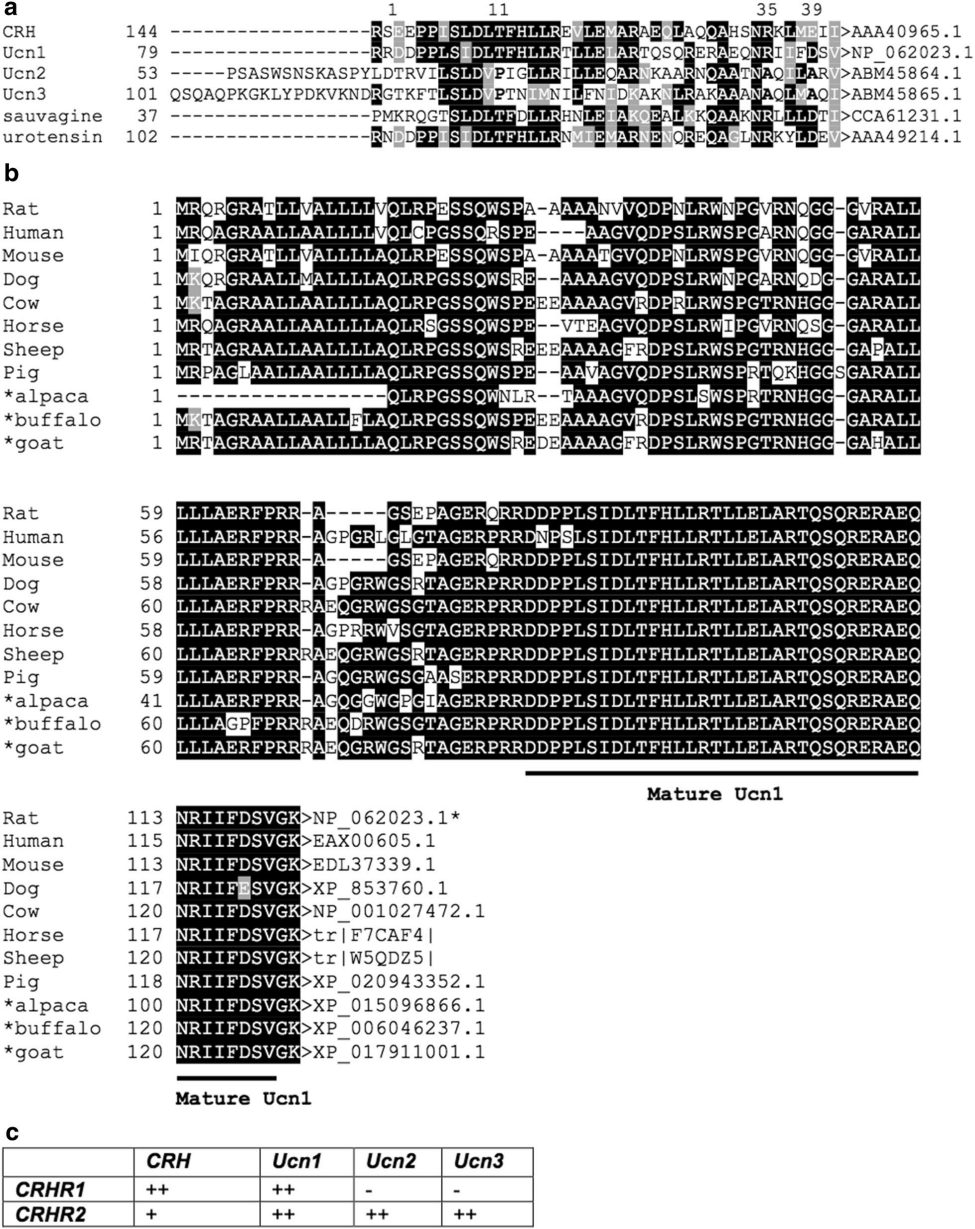
**a** Multiple alignment of the amino acid sequence of corticotropin-releasing hormone (CRH) and CRH-related peptides. The rat Ucn1 has 45% similarity to rCRH, Ucn2 has 34% similarity to CRH, Ucn3 has 26% similarity to CRH, frog Sauvagine has 50% similarity to CRH, fish Urotensin has 50% similarity to CRH. **b** Multiple alignment of the amino acid sequence of urocortin precursors from different species including some domestic animals. Rat mature Ucn1 has 99–100% similarity to dog, cow, horse, sheep, pig, alpaca, buffalo, and goat Ucn1. Ucn1 sequences have 99–100% similarity among different domestic animal species. Sequences were aligned using Clustal W then highlighted using BoxShade 3.21, which shades conserved residues according to whether they are completely conserved (black) or similar (gray). Hyphens indicate gaps in the sequence among the species. Asterisk indicates “predicted sequences”. **c** Binding affinity of CRHR-ligands. CRHR1 binds CRH as well as Ucn1, but not Ucn2 and Ucn3, whereas CRHR2 binds Ucns with a higher binding affinity than CRH. A proline residue at position 11 (bold letter) is found only in CRHR2-selective ligands, Ucn2 and Ucn3. These peptides also contain alanine residues at positions 35 and 39 (bold letters)

Urocortins (Ucns) belong to the corticotropin-releasing hormone (CRH) family, which includes CRH, fish urotensin I, frog sauvagine, Ucn1, Ucn2 (or stresscopin-related peptide), and Ucn3 (or stresscopin) [1–4]. This family is a critical regulator of the hypothalamic–pituitary–adrenal (HPA) axis, leading to subsequent release of adrenocorticotropic hormone and corticosteroids [5, 6]. These 38–41-amino acid peptides are structurally related and are highly conserved among different animal species [7, 8] (Fig. 1a). All peptides are encoded by separate genes. These peptides are found in the central nervous system (CNS) as well as in peripheral tissues including the digestive, cardiovascular, immune, reproductive, and endocrine systems [9–15]. The physiological effects of these peptides are mediated through seven transmembrane domain G-protein-coupled receptors (GPCRs): CRH receptor type 1 (CRHR1) and CRH receptor type 2 (CRHR2). CRHR1 and CRHR2 have differential binding affinities to each of the CRH family members. CRHR1 shows high affinity to CRH and Ucn1, but no appreciable binding affinity to Ucn2 and Ucn3. CRHR2 primarily binds to Ucn1, Ucn2, and Ucn3 with greater affinity than to CRH [3, 8, 16] (Fig. 1c). These receptors also have different expression patterns in the central and peripheral tissues [17]. CRHR1 is primarily expressed in the CNS and the anterior pituitary. The CRHR2 receptor is expressed primarily in extra-CNS sites. Two separate genes encode the CRH receptors [18]. In addition to binding to two receptors, CRH-related peptides also bind to CRH-binding protein (CRHBP) [19]. CRHBP, a 37-kDa glycoprotein, limits ligand availability [20].

Ucns have been shown to be associated with a variety of physiological functions in different animal species, including domestic animals. These functions include stress [21–24], feeding behaviors and energy homeostasis [25–31], immune responses [32], cardiovascular regulation [33–35], inflammation regulation [36], reproduction [37], and hormone release from the pituitary [38, 39].

Moreover, the presence of Ucns and their receptors was reported in several mammalian endocrine glands, thus suggesting a role for this peptide in regulating endocrine functions [13–15, 40, 41]. This review outlines existing knowledge on the expression and function of Ucns and their receptors in the mammalian endocrine system.

### Search strategy

This review is based on a search in PubMed (http://www.ncbi.nlm.nih.gov/pubmed) using the terms “urocortin, corticotropin-releasing hormone receptors, mammals, endocrine system”. The title and abstract of the obtained hits were evaluated and articles referring to urocortins in the endocrine system were obtained and assessed in detail. In addition, our own archives were used as a source of additional information. Our extensive experience with the expression of the urocortinergic system in the thyroid gland and testis of mammals was used to critically evaluate the literature.

## Review

### Urocortins and CRH-receptors: general structure and biochemistry

Ucn1 is a 40-amino acid neuropeptide cloned from rat brain tissue that has a 45% similarity with CRH and a 60% identity with urotensin I [8] (Fig. 1a). Ucn1 is composed of a single alpha helix structure [42]. Human Ucn consists of 124 amino acids, 80 of which form the “precursor peptide”. The amino acids Arg–Arg in position 81–82 and Lys-Gly in position 123–124 undergo proteolysis, constituting the site of detachment from the precursor and the C-terminal sequence, respectively. The mature Ucn1 peptide is generated by cleavage of 42 amino acids from the C-terminus followed by amidation to eliminate the C-terminal dipeptide Gly-Lys [43]. Since all the mammalian forms of Ucn1 discovered to date exhibit a high degree of sequence conservation, the functions of this peptide may be similar or identical across mammalian species. Ucn1 has been identified in humans, rats, mice, hamsters, and sheep [8, 43, 44] with high sequence homology [44]. In addition, the mature Ucn1 sequence is highly conserved in different domestic animal species (99–100% identity to rat, mouse or human, and among them) (Fig. 1b). The gene encoding Ucn1 is located on the short arm of chromosome 2 (2p23–p21). The genomic structure of the Ucn1 gene is similar to that of the CRH gene, with two exons and one intron. The entire precursor protein is located entirely on the second exon [8, 43]. Ucn1 binds both CRH receptors with high affinity [42].

Ucn2 is a 38-amino acid peptide related to CRH that shows homology with rat and human CRH (has about 34% similarity to rCRH and hCRH), hUcn1 (43%) and hUcn3 (37–40%) [3, 45]. Ucn2 and Ucn3, which were discovered during human genome mapping, are selective agonists of CRHR2 [45]. Human Ucn2 consists of two alpha helices, a short N–terminus and a long C-terminus, thus resulting in a helix-loop-helix structure. This structure is important for interaction with specific receptors [42]. The human gene encoding Ucn2 is located on chromosome 3 and the transcription of this gene is upregulated by glucocorticoids, suggesting that Ucn2 is involved in the stress response pathway [3, 46].

Ucn3 (also known as stresscopin) is composed of 38 amino acids and has about 20–40% similarity to h/rCRH and Ucn1. Both Ucn2 and Ucn3 share a sequence similarity of up to 40% [45]. The proline residue at position 11 and alanine residues at positions 35 and 39 are characteristic of CRHR2-selective ligands Ucn2 and Ucn3 [47] (Fig. 1a, c). Human Ucn3 consists of a small N-terminal helix and a long C-terminal alpha helix containing 20 residues. This type of helix-loop-helix is critical for interactions with specific receptors [42]. The links between the N- and C- terminal helices are thought to be crucial to ligand-receptor interactions [42]. The human gene encoding Ucn3 is located on chromosome 10 [48].

CRHR1 and CRHR2 are class B1 GPCRs consisting of seven transmembrane helices with an N-terminal extracellular ligand-binding domain (ECD). CRHR1, a 415-amino acid protein, shares 70% amino acidic sequence identity with CRHR2, but there is divergence at the N-terminal ECD. This is consistent with distinct pharmacological properties and agonist selectivity of both receptors. CRHR1 binds CRH and Ucn1, but not Ucn2 or Ucn3, with similar high affinity (Fig. 1c). On the contrary, CRHR2 binds all Ucns with higher binding affinity than for CRH, suggesting that these peptides may be its natural ligands [3, 8, 17, 18, 45, 48]. CRHR1 and CRHR2 are coded by two different genes. CRHR1 has been cloned in different mammalian species: humans, rats, mice, and sheep [18, 49–52]. The genes for human and rat CRHR1 contain 14 and 13 exons, respectively [53, 54], and produce several alternatively spliced isoforms. In humans, eight CRHR1 isoforms have been described [18, 47, 55, 56]. In rats, there are three CRHR1 isoforms [53]. In mice, there are four isoforms, equivalent to some human isoforms CRHR1alpha [50, 56]. The human CRHR2 gene contains 12 exons. Three major functional isoforms, alpha (411 amino acids), beta (438 amino acids), and gamma (397 amino acids), encoded by transcripts with alternative first exons [57] differ only in the N-terminal sequence comprising the signal peptide and part of the ECD (amino acids 18–108 of CRHR2 alpha). In mice, an mRNA splice variant of CRH-R2α (sCRH-R2α) was identified that encodes the receptor’s ligand-binding extracellular domain but terminates before the transmembrane domains. This variant was therefore predicted to serve as a secreted decoy receptor, mimicking the ability of CRH-binding protein to sequester free CRH [58]. This soluble variant is efficiently translated but not necessarily secreted [59].

CRHBP is a 37-kDa glycoprotein and the bound form of CRH and its related peptides is biologically inactive [60]. CRHBP, in this manner, may have a role in modulating the effects of CRHR-ligand binding [20, 61]. Similar to CRH and CRHRs, CRHBP is widely distributed throughout the brain [19]. In humans, but not known in rodents, CRHBP is also synthesized in the liver and placenta, and secreted into the general circulation. The presence of CRHBP in the CNS suggests that CRHBP may have additional roles. CRHBP also seems to facilitate some of the actions of CRH/Ucn1 in a CRHR2-dependent manner. In particular, CRH requires CRH-BP to potentiate NMDA receptors via CRHR2 in dopamine neurons [62]. Recently, Slater and colleagues demonstrated that CRF-BP acts as an escort protein for CRF2αR, facilitating its presence in the plasma membrane. These authors showed that CRF-BP physically interacts with CRF2αR in an isoform-specific manner, because no interaction was detected between CRF-BP and CRF2βR [63].

Activated CRHR1 and CRHR2 primarily signal by stimulating G protein (Gs) coupling, resulting in the induction of the cyclic adenosine monophosphate (cAMP)-protein kinase A (PKA) and the extracellular signal-regulated kinase-mitogen-activated protein kinase (ERK-MAPK) pathways. Activation of PKA leads to the phosphorylation of transcription factors like cAMP response element binding protein (CREB), which in turn increases the expression of downstream target genes [64]. CRHRs also interact with other G proteins, including Gq, Gi, and Gi1/2, thus activating phospholipase C variants (PLCs) and resulting in the activation of ERK1 and ERK2 and an increase in intracellular Ca^2+^ concentration [47, 65]. Many other regulatory systems seem to modulate CRHR activity but are not well understood and warrant further study.

### Hypothalamic–pituitary–adrenal and thyroid axis

Recent research has focused on possible roles played by Ucns and their cognate receptors CRHR1 and CRHR2, respectively on the HPA and thyroid axes. In particular, these studies demonstrated a relationship between their possible tissue distribution in these regions and their specific regulation in mammals.

Based on previous data reporting the role of CRH in multiple regulation mechanisms at the level of these regions and in consideration of the high correlation between Ucns and CRH, different studies have aimed to analyze the possible differences in the role of these peptides with respect to CRH. Since the HPA axis plays a pivotal role in regulating fundamental processes of all living organisms [66], the participation of these peptides in different activities of this system may be significant. It is well known that the HPA axis not only involves the functions of three endocrine glands (hypothalamus, pituitary, and adrenal glands), but also is widely involved in different physiological processes such as regulation of the stress response [67], digestion, immune function, behavior, sexuality, and energy storage and expenditure.

Similarly, the HPT axis plays a pivotal role in stimulating the normal secretion of thyroid hormone and thus contributes to maintain cardiovascular, bone, and liver function, and energy homeostasis [68]. Such complex activities of both the HPA and HPT axis are deeply controlled by complex regulatory circuits that permit communication between different regions, either through cell-to-cell communication (paracrine signaling) and within the same cell communication (autocrine signaling). In this context, studies concerning Ucn distribution at the level of these endocrine axes in domestic animals (Table 1) indicate their possible involvement in regulating numerous biological functions. These findings could clarify previous data reports often overshadowed by studies on CRH and its analogues, thus leaving aside many other angles of specific research.

**Table 1:** Expression of urocortins (Ucns) and their relative receptors, CRHRs, in endocrine system tissues of domestic animals

Endocrine system	Animal species	Ucn1	Ucn2	Ucn3	CRHR1	CRHR2	References
HPA	Cat	+	n.d.	n.d.	n.d.	n.d.	[67]
	Bird	n.d.	n.d.	+	n.d.	n.d.	[153]
	Cow	+	n.d.	n.d.	+	+	[13]
	Sheep	+	n.d.	n.d.	n.d.	n.d.	[39, 68]
HPT	Cow	+	n.d.	n.d.	+	+	[15]
	Horse	+	n.d.	n.d.	+	+	[14]
Female gonads	Pig (ovary-CL)	+	n.d.	n.d.	+	+	[117]
	Pig (ovary)	+	n.d.	n.d.	+	–	[117]
	Sheep (placentome)	+	n.d.	n.d.	+	n.d.	[126]
Male gonads	Dog (testis)	+	n.d.	n.d.	+	+	[104]
	Alpaca (epididymis)	+	n.d.	n.d.	+	+	[111]

*HPA* hypothalamic–pituitary–adrenal axis, *HPT* hypothalamic-pituitary-thyroid axis, *CL* corpus luteum, *n.d.* not determined

Ucn1, the most-studied Ucn, has been characterized in terms of its localization in different domestic animal species, also focusing on its possible role in the CNS. In particular, Ucn1 identification in cats, particularly within the Edinger-Westphal (EW) nucleus [69], which differs from that observed in ovine species, where the peptide was observed in the hypothalamic region, could suggest a particular role in this area as well as a specific binding activity towards CRH receptors, if compared with its homolog human Ucn1 [70].

Considering that in rats, Ucn1 localizes not only to the EW [71, 72] but also to lateral superior olivary (LSO) and supraoptic nuclei [73], the idea of a different modulation in Ucn1 activity in domestic animal species should potentially be considered. The presence of Ucn1 in cats at the EW nucleus suggests a specific and confined role of this peptide, although the functional studies on this topic are limited. Moreover, it should be considered that the EW nucleus and the location of Ucn cell population differ among animal species (particularly between cats and macaque monkeys), although, as described for monkeys, in some cases, the distribution of the perioculomotor (pIIIU) cell population is likely to overlap with that of C- and S-group motorneurons that supply non-twitch muscle fibers in the extraocular muscles [69]. This organization of Ucn cell populations and their projections suggest possible functional implications of Ucn1 and of the use of specialized neurotransmitters, that currently remain an open and testable hypothesis.

The direct activity of Ucn1 on ACTH release from the pituitary has been assessed in a complex research project in sheep focused on evaluating the endocrine effects of Ucn1 in experimental heart failure [67].

In contrast, in several studies conducted on rat species, the presence of Ucn1 mRNA in the brain and pituitary and the biological effects of Ucn1 after its intracerebroventricular (ICV) or intravenous (IV) administration suggest its complex role in regulating the HPA axis [38, 73].

In 2011, Ucn1 as well as its relative receptors CRHR1 and CRHR2 were isolated and characterized in the bovine adrenal gland, showing their particular distribution in both adrenal cortex and medulla [13]. Such results, confirmed by the use of histological and biochemical techniques, permit us to speculate about the role of Ucn1 in the intra-adrenal CRH-based regulatory system to be achieved by an autocrine mechanism [13]. Moreover, other data demonstrating the role of Ucn1 in adequate control of steroid secretion come from studies in lactating dairy cows with or without ovarian follicular cysts [74]. Together, these data confirm previous findings obtained from studies using knockout (KO) mouse models lacking Ucn1, indicating cellular hypotrophy of the outer adrenal cortex and lower expression levels of Cyp11b2 [75]. However, Ucn1 knockout mice show no alterations in HPA axis activity [76, 77]. In addition, in mice deficient in Ucn2 or Ucn3, as single, double, or triple knockout in combination with Ucn1, alterations of HPA axis functions have not been observed [78–80]. The use of these models is undoubtedly important to clarify some metabolic functions of Ucns.

Studies conducted by directly administering Ucn1 have contributed to a better knowledge of the mechanisms exerted by Ucn1 and, in some cases, on its receptors CRHR1 and CRHR2 on the HPA axis of domestic animals (Table 2). In particular, Parrott et al. [81] demonstrated that Ucn1, when administered by intracerebroventricular injection (ICV), induced an increase in cortisol secretion similar to that induced by CRH, albeit the latter showed a higher stimulatory activity. Similar results were observed considering behavioral activation parameters (such as changes in posture and orientation, and engagement in vigorous oro-nasal activity). The interpretation of these results should take into account that, in contrast to humans and rats, for which the CRHR distribution in the CNS has been identified [82], pig Ucn1 CRHR2 has not yet been discovered. Therefore, considering that Ucn1 activity mostly occurs through CRHR2, the different selectivity of CRHRs varies with animal species [83]. This last consideration could be important to evaluate the effect of Ucn1 in sheep, even if the recorded effects were similar between Ucn1 and CRF in this species [84].

**Table 2:** Effects of administering urocortins (Ucns) and their relative receptors, CRHRs, on the hypothalamic–pituitary–adrenal axis of domestic animals

Animal species	Treatment	Ucn1	Ucn2	Ucn3	CRHR1	CRHR2	Effects	References
Bird	ICV	n.d.	n.d.	+	n.d.	n.d.	↑ Ghrelin	[154]
Ewe	ICV	+	n.d.	n.d.	n.d.	n.d.	↓ Food intake ↑ GH, LH, cortisol, leptin	[84]
Gilt	ICV	+	n.d.	n.d.	n.d.	n.d.	↓ LH and food intake ↑ Cortisol and ACTH	[85]
Horse (pony)	ICV	+	n.d.	n.d.	n.d.	n.d.	↑ Cortisol ↓ Food intake	[155]
Pig	Central injection	+	n.d.	n.d.	+	n.d.	↑ Cortisol	[81, 156]
Sheep	ICV	+	n.d.	n.d.	n.d.	n.d.	↑ Urine excretion ↓ Food intake	[157]

*ICV* intracerebroventricular, *n.d.* not determined, *GH* growth hormone, *LH* luteinizing hormone, *ACTH* adrenocorticotropic hormone

The possibility of Ucn1 to exert its activities by binding to its specific CRHR2, which has not yet been identified in all tissues in different animal species, permits speculation that Ucn1 exerts its activity in a broad range of areas. In particular, the integrative role of Ucn1 as a mediator in the interrelations between systems of appetite regulation, reproduction, growth, and metabolic balance has been evidenced in a study in gilts [85].

More recently, expression of Ucn1, CRHR1, and CRHR2 has been investigated in both equine and bovine thyroid glands [14, 15]. Such studies, supported by the use of multiple techniques (immunohistochemistry, western blotting, and RT-PCR), have greatly contributed to knowledge on the potential role of Ucn1 in this gland in domestic animals. In particular, in horses, immunohistochemical results revealed for the first time that Ucn1 expression specifically localizes in thyroid follicular cells, although CRHR2 was detected in both parafollicular and C-cells [14]. Results from bovine species [15] revealed a different distribution of Ucn1 and CRHR2 at the cellular level, confirmed by immunoreactivity in both follicular and parafollicular cells. Conversely, CRHR1 immunoreactivity was concentrated in the smooth musculature of blood vessels in both animal species, thus suggesting a potential role in modulating the thyroid gland blood flow, as previously reported in rats and mice [86–88].

In particular, western blotting showed that Ucn1 presents as a band corresponding to a molecular weight approximately of 16 kDa, comparable with the mammalian Ucn precursor (122-amino acid protein) [8, 13, 43]. Its characterization suggests a role in calcitonin secretion via CRHR2 in an autocrine/paracrine manner, probably adjuvated by other CRHR2 ligands, such as Ucn2 and Ucn3. All these findings suggest a possible role of follicular cells in regulating iodine uptake, supported by specific local blood flow. Such data confirm previous results by studies on pathological conditions in humans [89]. In particular, results from a case of multiple endocrine neoplasia type II showed the presence of cells highly expressing CRH, Ucn1, and Ucn3.

Together, these data indicate that CRH and Ucns play a role in secreting ACTH and glucocorticoid hormones. However, further studies are needed to investigate the possible involvement of Ucns in thyroid hormone synthesis. Regarding the parathyroid, only a functional study by Asakawa et al. [90] demonstrated that the influence of parathyroid hormone-related protein on food intake by gastric emptying is related to Ucn2 and Ucn3 expression.

More recently, new promising perspectives regarding the involvement of Ucns (particularly Ucn2 and Ucn3) in the field of stress pathophysiology have been shown. This area of research is attractive considering that different forms of stress (physical and psychological) interfere by multiple mechanisms (central and peripheral) on several body activities (metabolism, reproduction, behavior, host defense, etc.). In this regard, although several studies on Ucn involvement in stress have been conducted in laboratory animals [78, 91–94], more research has been focused on domestic animals. Moreover, it should be taken into account that Ucns and CRH have been found in brain areas known to be important not only for body fluid and electrolyte homeostasis but also for behavior. In addition, Ucns (Ucn2 and Ucn3) seem to be particularly involved in regulating mammalian social behavior via activation of CRFR2 [95].

Some reports, based on results showing that stress can cause an increase in Na intake similar to the effect obtained by peripheral Ucn administration in several different species, including sheep [96], rabbits [97], rats [98], and mice [99, 100], have stimulated interest on this research area. In sheep and rats, the appetite-related effects appear to occur via ACTH on adrenal gland hormones, because it can be prevented by adrenalectomy [96, 98]. On the other hand, ACTH can cause an increase in Na intake in adrenalectomized wild rabbits [97]. Weisinger et al. [101] demonstrated that Ucn, administered by ICV infusion, inhibited Na intake in sheep. In particular, Ucn inhibited not only need-free Na intake but also the high Na intake induced by peripheral ACTH, further indicating a definite inhibitory action. Taken together, these data suggest that Ucn and CRH can influence some, but not all, activities involved in Na appetite—possibly only those initiated by corticosteroids. The mechanism remains to be clarified completely, because the brain nuclei that mediate Na appetite in sheep are not fully known. Presumably, other physiological processes entrained by stress that are important in determining the outcome on Na appetite need further investigation. In the meantime, studies in rats indicate that steroid-induced, but not Na depletion-induced, Na appetite is mediated in the medial amygdala [102], a brain area beyond the blood–brain barrier. Moreover, experiments analyzing the specific activity of urocortin with respect to the well-known CRH, demonstrated that the two peptides could have both central and peripheral actions.

More recently, in a study regarding chronic social stress (CSS) in pigs that analyzed major factors driving gastrointestinal (GI) pathophysiology, Ucn2 mRNA was found to be up-regulated in the colon of pigs under CSS. These data have further confirmed the peripheral action of Ucn [103].

The data observed in domestic animal species could represent useful supporting evidence to that obtained in other species such as mice and rats, thus opening a new door to analyze the key role of Ucns related to biochemical, hormonal, and behavioral alterations in response to stress. The possible involvement of Ucns in the effects of stress should be of particular interest in farming animal species where specific conditions (food and/or water restriction or deprivation, social interaction, environmental conditions, animal handling, etc.) can alter the normal body homeostasis, which affects various physiological aspects and thus their performance. This concept also deserves further study, since Ucns may be involved in stress-related psycho-pathologies (depression and anxiety) [60], and discriminating between the specific action of Ucns might help determine their differential roles in regulating the stress response.

### Gonads and reproductive system

Studies of the Ucns within the endocrine organs helped define the localization and physiological roles of these peptides and their receptors in the gonads and in diffuse cells of the reproductive system. The gonads play a pivotal role in regulating gametogenesis and steroidogenesis, thereby influencing reproductive performance. In this context, investigating Ucns in domestic and livestockin both healthy and pathological conditions might be relevant for improving knowledge regarding reproduction, particularly in clinical practices. Few studies regarding the presence of Ucns and CRHRs in the male gonads of domestic animals have been performed, and one of these studies was conducted by our research group [104] (Table 1). We described Ucn1 and CRHRs in the testes of normal and cryptorchid dogs by means of immunohistochemistry, western blot, and real-time RT-PCR. Particularly, Ucn1 and CRHR2 were observed in the tubular and interstitial compartments of normal and cryptic gonads, leading us to hypothesize that these substances may play a role in regulating mitotic and apoptotic events occurring during spermatogenesis and in regulating steroidogenesis by an autocrine/paracrine mechanism. On the other hand, the distribution of CRHR1 in the muscular cells of blood vessels within the normal and cryptic gonads suggests a role of Ucn1, via CRHR1, in regulating canine testicular blood flow. The most interesting finding of this research was a decrease in Ucn1 and CRHR2 mRNA levels in cryptic canine testis, suggesting that these peptides play a role in preventing neoplasia. This last aspect deserves particular attention, as the Ucns could represent targets for possible pharmacological or therapeutic approaches for cryptorchidism, suggesting modulation of the main testicular functions, and consequently, reproductive performance.

These hypotheses are based on published data in humans and rodents. The only evidence of Ucn1 and CRHRs in the human testis was demonstrated by Tezval et al. [105] who detected a possible role of these peptides in the pathophysiology of germ cell differentiation and division in normal adult and fetal testicular germ cell distribution. Most studies regarding Ucns and their receptors in the male gonads were performed in rodents, with controversial results. Particularly, Ucn1 located in mouse spermatozoa had inhibitory effects on T-type calcium channels in mouse spermatogenic cells, sperm motility, and progesterone-evoked sperm acrosome reaction, indicating that inhibition of Ca^2+^ channels might induce inhibitory effects of Ucn1 on male reproductive functions [106]. In rat testis, intragastric alcohol injection significantly increased mRNA levels of testicular Ucn1, but not Ucn2, Ucn3, CRHR1, or CRHR2 [12]. Although McDowel et al. [107] demonstrated the ability of Ucn1, by binding CRHR1, to elevate steroidogenic gene expression in rat and mouse fetal Leydig cells, in the rat testes, this substance seemed to interfere with Leydig cell activity by inhibiting human chorionic gonadotropin-stimulated steroidogenesis in primary adult rat Leydig cells [108]. However, Ucn1 may play a cytoprotective role in the germ cells from rat testis in response to ischemia–reperfusion injury through activating major anti-apoptotic proteins, as well as by activating the MAPK signaling pathway [109]. Ucn-mediated steroidogenic/steroidolytic mechanisms could represent a starting point for studying these substances in the reproductive sphere, regarding the health and disease of domestic animals. It would be interesting to deepen our understanding about the implication of these peptides in stimulating or inhibiting steroidogenic enzymes and subsequent repercussions on male fertility of domestic and farming animals, which would have great relevance for clinical practices, and eventually for therapeutic approaches. Currently, these remain only hypotheses.

Not only the male gonads, but also the diffuse “endocrine” cells, localized in the male genital tract participate in regulating reproductive functions. Particularly, Ucn1 and CRHRs were also detected in some “endocrine” cells of male reproductive organs, such as the epididymis of rats [110] and alpacas (*Vicugna pacos*) (Table 1) [111], as well as normal, hyperplastic, and neoplastic prostates in humans [112–114]. In the alpaca epididymis, Ucn1 and CRHR2 were found in the luminal portion of the epididymal principal cells, while CRHR1 was found in the fibromuscular cells encircling the epididymal tubules. Specifically, it has been hypothesized that Ucn1, via CRHR2, may be involved in absorptive and secretory activities of the luminal compartment of the epididymis. Moreover, via CRHR1, Ucn1 can modulate the contractility of this organ. On this basis, it might be hypothesized that Ucns in the epididymis could be involved in regulating male fertility, acting on the maturation and storage of spermatozoa and their transition throughout this organ. As in the human prostate, Ucns and their receptors might play multiple roles in regulation of prostate pathophysiology in domestic and farming animals [112–115]; therefore, future studies should investigate these peptides in other animals.

The presence of Ucns in the female gonads of domestic animals is unknown, while several studies were performed on primates, humans, and rodents. To our knowledge, the only evidence of Ucns in the female reproductive system of domestic animals involves the porcine ovary, in which these peptides seemed to be mainly implicated in regulating steroidogenesis [116, 117] (Table 1). Sakumoto et al. [116] provided evidence for the existence of mRNAs encoding CRHR1 in the porcine corpus luteum (CL) throughout the estrous cycle. mRNA coding for CRHR1 was expressed at the highest levels in the CL of the regressed stage. CRH, via CRHR1, inhibited progesterone production by luteal cells while it did not affect estradiol-17β and prostaglandin F2α in this species. This research group suggested that CRH plays one or more roles in regulating porcine CL function during the estrous cycle, especially at luteolysis (Table 1). Another important factor involved in ovarian function is microRNA-375 (miR-375). For this purpose, Yu et al. [117] investigated the relationship between miR-375 and CRH signaling molecules in the porcine ovary. PCR results showed that miR-375 and CRHR1 are expressed in the porcine ovary, whereas CRHR2 was not observed. These two factors seemed to be co-localized in ovarian granulosa cells, whereas CRHR1 was detected also in oocytes. The overexpression of miR-375 in cultured granulosa cells inhibits estrogen production. Thus, miR-375 is a key factor in regulating estrogen synthesis by mediating the CRH signaling pathway. The findings related to these substances in the female gonad of domestic animals are limited to CRH and its binding to cognate receptors.

In the ovarian CL of primates, Ucn1, Ucn2, CRHRs, and CRHBP were described [118, 119]. In primate species, CRH/Ucns promoted luteal development and function but not the ovulatory process. In the human ovary, immunoreactive Ucn1 expression was found in luteinized granulosa and in theca cells of the functioning and the regressing CL, and CRHRs were higher expressed in luteinized thecal cells of regressing CL. These findings suggested that Ucns and CRH were involved in suppressing steroidogenesis [120]. mRNAs encoding both CRHRs have been found during the late follicular phase in granulosa cells, interstitial cells in the ovarian stroma, and to a greater extent in theca cells of dominant follicles [120, 121]. The increased expression of CRHR1 observed in mature human follicles implied a role of CRH-related peptides in follicular maturation [122]. Although initial studies failed to detect Ucn3 in the human ovary, it was later found in human granulosa-lutein cells of infertile women undergoing in vitro fertilization [119] and it was hypothesized to have an inhibitory effect on progesterone production [123]. Mouse models of both sexes overexpressing Ucn2 exhibited significantly higher plasma Ucn2 levels and Ucn2 expression levels in the adrenals and ovaries [124]. Ucn2 overexpression in the ovaries decreased steroidogenesis and reduced the number of follicles that had undergone ovulation, which was not associated with reduced fertility. Ucn2, Ucn3, and CRHR1 expression was also identified in rat ovaries [45, 125]. The selectivity of the ovarian CRHR1 gene expression may suggest a biological action of CRH during the ovulatory event within the rat gonadal life cycle, in both control and stressful conditions. In line with what is described in primates and rodents, it might be hypothesized that even in domestic animals, CRH family proteins could modulate ovarian steroidogenesis and ovulatory events, consequently affecting reproductive function. Currently, these are only hypotheses that would require investigation.

Ucns and CRHRs were also described in the placenta and uterus, where they are involved in several endocrine processes. There is only one published description of Ucns in the placenta of domestic animals. Particularly, CRH and Ucn1 have been identified in the ovine placentome by immunohistochemistry [126] (Table 1). Most of the information related to this topic is limited to humans and rodents. Specifically, Ucn1, Ucn2, and Ucn3 were described in the human placenta and fetal membranes [127]. Ucn2 and Ucn3, found throughout human gestation in both the maternal and fetal membranes and decidua, have been described as regulators of placental vascular endothelial behavior and tone [128]. The different distribution of these peptides in the human gestational tissues supports the hypothesis that they may serve different functions during pregnancy, although published data are still conflicting [129–131]. Similar profiles of Ucn2 mRNA and protein expression increase were assessed in mice during gestation, in which Ucn2 seemed to be implicated in the process of parturition [132]. In human trophoblast cell cultures, the endocrine actions of Ucn2, via CRHR2, include the regulation of aromatase activity and estrogen stimulation [133], and inhibition of placental 15-hydroxyprostaglandin dehydrogenase [134]. The immunoregulatory actions of Ucn2 also include amplification of placental lipopolysaccharide-induced [135] secretion of tumor necrosis factor (TNF)- and IL-10 [136]. These findings suggest that Ucn2 contributes to placental regulation in pregnancy and parturition events.

Concerning the diffuse endocrine cells in the female reproductive system, Watanabe et al. [31] detected Ucn2 in endometrial glands and epithelial cells of the rat uterus. mRNA levels of Ucn2 and CRHR2 in the uterus of mature rats were significantly higher in the diestrus phase than those in the proestrus phase, while plasma estrogen concentrations were significantly lower in the diestrus phase than in the proestrus phase. The uterine Ucn2/CRHR2 system might communicate with the estrogen system and may have regulatory roles in the estrous cycle. Ucn2 mRNA is strongly expressed in the uterus during the proliferative phase in healthy women, and this expression is abolished in patients with endometriosis [136]. In summary, the effects of Ucns on placental regulation, and in pregnancy and parturition events described in rodents and primates could be extended to farming animal species. Cows are frequently affected by retained fetal membranes, which represents a major problem in reproduction.

### Digestive system

Extensive data on the distribution of Ucns and CRHRs in other organs suggest that these peptides have a more significant role in regulating the diffuse endocrine system. In this context, the expression of Ucns and their receptors as diffuse endocrine cells has mostly been found in the gastrointestinal tract of humans and rodents [45, 137–146]. Few papers described the expression of Ucns and their receptors in the diffuse endocrine gastrointestinal system of domestic animals. Particularly, Ucn1, CRHR1, and CRHR2 were detected in the distal colon fetuses from ovine species, where they were expressed in many sites and particularly in diffuse endocrine epithelial cells. Particularly, the co-localization of muscarinic receptor subtype M4 and weak CRHR2 signals on epithelial cell surface of colon fetuses suggests that these two receptors may modulate ion transport functions of the M3 subtype receptor. Strong CRH signals in the cytoplasm of luminal colonocytes and moderate Ucn1 signals in epithelial cells indicate that both neuropeptides may regulate epithelial M3 receptor-mediated ion transport under stress conditions via CRHR2. In adult animals, stress is known to induce epithelial dysfunction (breaking of the epithelial barrier) including mucin secretion and transmucosal fluxes of macromolecules and electrolytes [147, 148]. Reports describing the localization of CRH and Ucn1 in enterochromaffin cells and enteric neurons of rodents and humans indicate that endocrine and neural tissues may function as local sources for both neuropeptides to mediate gastric inhibitory and colonic stimulatory motility effects via autocrine or paracrine mechanisms [137, 138, 144].

In the digestive apparatus of mammals, the pancreas is a glandular organ whose endocrine cells are organized in small clusters, the islets. UCN3 is strongly expressed in mammalian pancreatic beta cells and has been shown to stimulate insulin secretion. The expression pattern of UCN3 differs considerably between rodent and primate islets. While mouse UCN3 is restricted to beta-cells [149, 150], its expression in macaque (*Macaca nemestrina*) and human islets extends to the alpha-cells [149]. Consistent with this notion, Li et al. [150] have reported that exogenous UCN3, acting through CRHR2, stimulates insulin and glucagon secretion, particularly in the presence of nutrient excess. In rat and mouse insulin-secreting cell lines (INS-1 and MIN6), the expression levels of CRHR1 are notably higher than levels of CRHR2, a balance that can be overturned by exposure to glucocorticoids (GCs) [41]. The plasma glucose concentration and pancreatic insulin and glucagon secretion are relevant in various physiological states of domestic animals (i.e. lactation in dairy cattle). Several neuroendocrine substances are involved in these states.

### Endothelium

Situated at the interface between the blood and the inner wall of the vasculature, the endothelium is currently considered as not only a protective barrier, but also a central player that maintains cardiovascular homeostasis by secreting different substances. Ucn is one of these substances and has a dual effect on endothelium function [151, 152].

The endothelium, which also exhibits strong CRH-R2 expression, may contribute significantly to Ucn-induced vasodilation in some situations; deendothelialization was reported to reduce the vasodilatory action of Ucn1 by as much as 50% in rat aorta [53]. Studies in rat and pig coronary arteries [30, 69, 70] and human internal mammary arteries [73] indicate that Ucn1 endothelium–dependent dilation involves both nitric oxide (NO) (via second messenger cyclic guanosine mono- phosphate), prostaglandins, and barium-sensitive and calcium-activated potassium channels. Ucn1-related vasodilation of renal arteries is reported to be mediated by the production of cAMP, synthesis and release of sarcoplasmic Ca^2+^ as well as NO, and activation of potassium channels [74, 75].

## Conclusions

The widely documented distribution of Ucns in different tissues of domestic animals provides evidence for the important biological role of these peptides in regulating bodily homeostasis. However, considering that more recent studies conducted on domestic animal species have used Ucn administration to evaluate its effects, the results obtained from this approach do not always give a complete and clear pattern of activity. Indeed, it is important to analyze the convolution of all the possible conditions causing endogenous ligand release, which involves an orchestrated complex of stress mediators—neurotransmitters, neuropeptides and steroids—that are released throughout the entire brain.

**Fig. 2:**
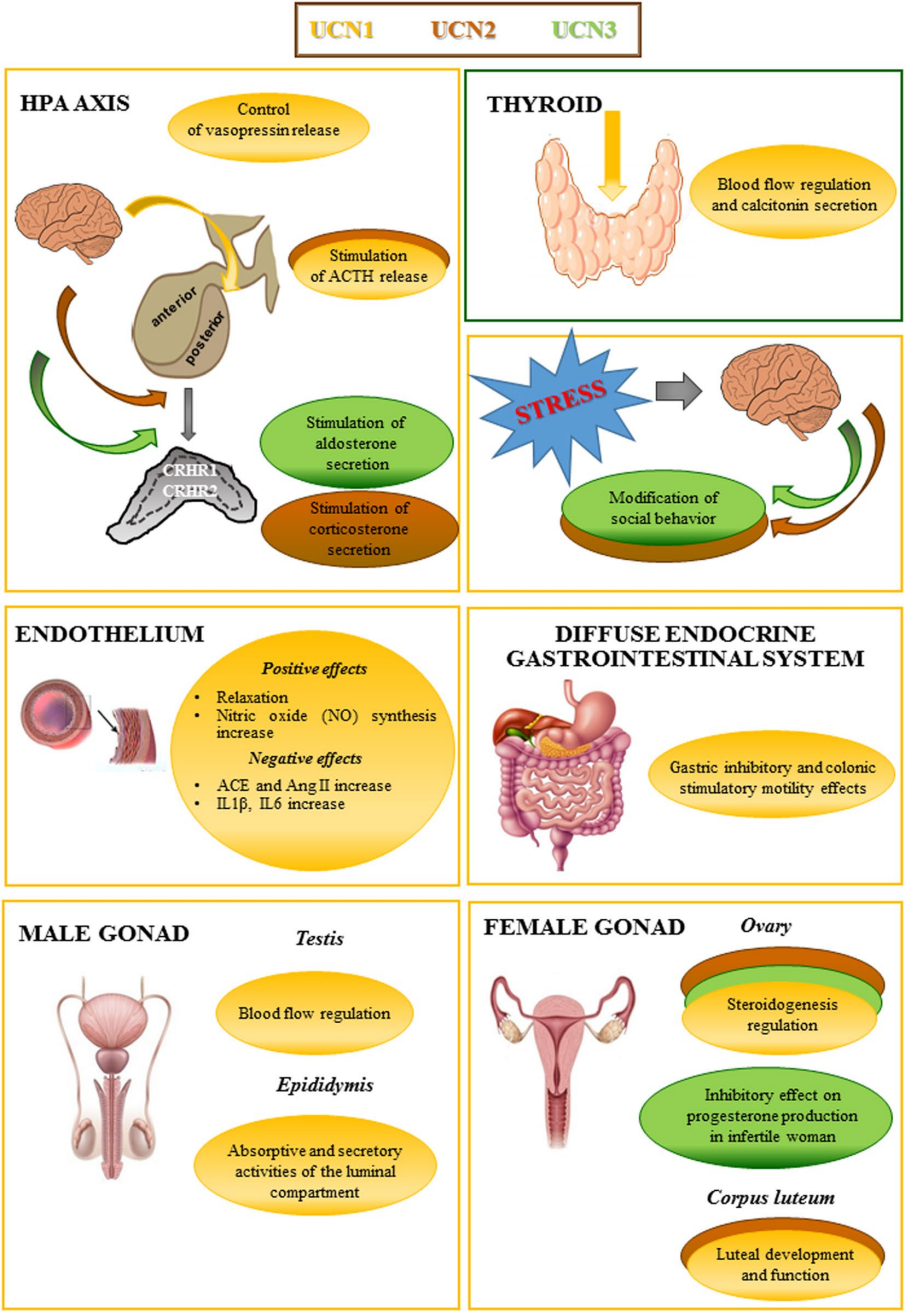
Illustration of the principal involvement of urocortins (Ucns) in regulating different endocrine organs

Moreover, considering the important involvement of Ucns in different physiological and pathophysiological conditions related to the different endocrine glands (Fig. 2), they may be applied clinically to treat different diseases. This possibility is also derived from the wide spectrum of action shown by these neuropeptides at the central and peripheral level. Thus, future investigations addressing these aspects certainly could help fully characterize these peptides.
